# Influence of Canning and Storage on Physicochemical Properties, Antioxidant Properties, and Bioactive Compounds of Apricot (*Prunus armeniaca* L.) Wholes, Halves, and Pulp

**DOI:** 10.3389/fnut.2022.850730

**Published:** 2022-05-10

**Authors:** Nusrat Jan, Sadaf Anjum, Sajad Mohd Wani, Sajad Ahmad Mir, A. R. Malik, Sajad Ahmad Wani, Dina S. Hussein, Rabab Ahmed Rasheed, Mansour K. Gatasheh

**Affiliations:** ^1^Division of Food Science and Technology, Sher-e-Kashmir University of Agricultural Sciences and Technology of Kashmir, Srinagar, India; ^2^Department of Food Science and Technology, University of Kashmir, Srinagar, India; ^3^Division of Fruit Science, Sher-e-Kashmir University of Agricultural Sciences and Technology of Kashmir, Srinagar, India; ^4^Department of Food Technology, Islamic University of Science and Technology, Awantipora, India; ^5^Department of Chemistry, College of Sciences and Health, Cleveland State University, Cleveland, OH, United States; ^6^Histology and Cell Biology Department, Faculty of Medicine, King Salman International University, South Sinai, Egypt; ^7^Department of Biochemistry, College of Science, King Saud University, Riyadh, Saudi Arabia

**Keywords:** apricots, canning, storage, antioxidants, bioactive compounds, HPLC

## Abstract

This study aimed to examine the effect of canning and storage on physicochemical, mineral, and antioxidant properties and phenolic composition of apricot wholes, halves, and pulp. The findings for physicochemical properties revealed that the total soluble solids, titratable acidity, total sugars, and ascorbic acid were found higher in apricot pulp (37.15, 1.39, and 20.74% and 7.21 mg/100 g FW, respectively) followed by apricot wholes and halves throughout the storage period. The remarkable contents of potassium, phosphorous, zinc, copper, iron, and manganese were found in the apricot pulp which revealed that canning and storage slightly affected the mineral composition. Bioactive substances were identified and quantified by reversed-phase high-performance liquid chromatography, which indicated a higher presence of chlorogenic acid (34.45 mg/kg FW), quercitin-3-glucoside (16.78 mg/kg FW), neochlorogenic acid (26.52 mg/kg FW), gallic acid (5.37 mg/kg FW), kaempferol (14.22 mg/kg FW), ellagic acid (6.02 mg/kg FW), procyanidin B_2_ (8.80 mg/kg FW), and epicatechin (9.87 mg/kg FW) in apricot pulp followed by apricot wholes and halves throughout the storage period. The total phenolic content was found highest in apricot pulp (13.76 GAE mg/100 g FW) followed by wholes (8.09 GAE mg/100 g FW) and halves (6.48 GAE mg/100 g FW) which decreased significantly throughout the storage period. Antioxidant properties were assessed by DPPH, ABTS^+^, MCA, and BCBA, which were found higher in the apricot pulp (92.23 TEAC μg/g DW, 92.33 TEAC μg/g DW, 33.80 TEAC μg/g DW, and 68.40 TEAC μg/g DW, respectively) that is correlated with the higher presence of bioactive compounds. Thus, apricot pulp containing excellent sources of nutrients, minerals, phytochemicals, and antioxidant components could be used for consumption purposes that provide nutraceuticals and antioxidants globally.

## Introduction

The imbalance between antioxidants (AOXs) and free radicals causes oxidative stress (1). Free radicals are molecules that have an unpaired electron (2). These molecules are extremely reactive and play a critical function in cell physiology, including regulation of life cycle, migration, development, activation of second messengers, signaling pathway stimulation, and AOX response triggering (3). Various disorders including cardiovascular disorder, hypertension, cancer, diabetes, atherosclerosis, and arthritis are linked to oxidative stress (3, 4). Fruits and vegetables (F&Vs) are natural sources of AOXs such as phenolic acids, including hydroxycinnamic acids (5) and hydroxybenzoic acids (6); flavonoids, including flavones (7), flavanols (8), flavonols (9), and flavanones (10); vitamin C (11, 12); and pigments like xanthophylls (13), betalain (14), carotenoids (15), chlorophyll a, chlorophyll b (16), and beta-carotene (17) that have high radical quenching ability (18). These are also the essential sources of minerals, such as macroelements (e.g., K, Ca, Mg, P, and S) (19) and microelements (e.g., Fe, Cu, Mn, Zn, Na, Mo, and B) (20), proteins (21), carbohydrates (22), and vitamins (23) for human nutrition. These chemicals are crucial in the production of functional foods. Fruits can be regarded as natural materials for preventing many diseases in humans, as they may lessen the risk of numerous age-related degenerative disorders (24).

Apricot (*Prunus armeniaca* L.) fruit is consumed as a fresh, dried, and processed product and has positive effects on human nutrition and health (25, 26). It is an essential food source for humans as it provides an optimal mix of bioactive phytochemicals, minerals (especially K, Fe, Mg, and P), fibers, sugars, and vitamins including A, C, riboflavin, thiamine, pantothenic acid, and niacin (27, 28). Nutritionally, apricots are chief sources of AOXs, carotenoids, and phenolics which are significant phytochemicals for their biological importance. The main sugar components are sucrose, glucose, and fructose (29). Lutein, β-carotene, γ-carotene, and β-cryptoxanthin are the most prevalent carotenoids contained in apricot fruit (28). The main phenolic substances present in apricots are catechin, neochlorogenic acid, epicatechin, and chlorogenic acid (30, 31). These compounds are antibacterial, anti-inflammatory, antimutagenic, and anti-allergic, and they have the potential to prevent cancer and coronary heart disease (32, 33). Therefore, apricots have been claimed to be a functional food for improving health and quality of life by boosting the body's defensive mechanism against free radicals, delaying aging, and protecting the body from diseases (25). However, apricots are climacteric in nature and highly perishable stone fruit; various postharvest conditions such as rapid ripening, weight loss, decay, and tissue softening limit their shelf life. In order to reduce its postharvest loss and avail its benefits round the year, apricots are processed in a variety of ways that include drying, freezing, and canning.

Among the successful operational processing techniques, canning is the most common method of food preservation which includes immersing food in acid brine, heating it, exhausting it, and closing it with hermetic seals. Although this technique ensures food safety, it can have an impact on the mechanical characteristics, taste, color, and nutritional quality of food (34). Furthermore, various changes occur in the phytochemical, nutritional, and AOX values during the processing of fruits. Several researchers have reported a decrease in phytochemical content during processing (35–37), while others have reported an increase (28, 38) or no change in phytochemicals (39). Conditions during the storage of food products like storage duration, light, and temperature affect phytochemical retention. Therefore, this study was undertaken to investigate the influence of canning and storage on physicochemical, mineral, AOX characteristics and polyphenolic substances of apricot wholes, halves, and pulp.

## Materials and Methods

### Raw Materials

Commercial apricot fruits (*Prunus armeniaca* L.) were procured at the maturity stage in the month of July from ICAR-Central Institute of Temperate Horticulture, Srinagar, Jammu and Kashmir, India. Fruits having bruising, mechanical damage, or diseases were separated. Only those apricot fruits that were uniform in size, shape, and color were selected for the investigation. Standards for high-performance liquid chromatography (HPLC) were procured from Sigma-Aldrich (Steinheim Germany). All other chemicals and reagents used in the experiment had an analytical grade and were obtained from HiMedia India.

### Canning and Storage of Apricot Wholes, Halves, and Pulp

Apricots were canned following the technique illustrated by Wani et al. (28) with minor modifications. The apricots were categorized into three lots, each containing 100 apricots. One lot was canned whole, the second lot was canned in halves after the stones were removed, and the third lot was canned as pulp. Bajaj pulper was used to turn the apricots into fine pulp after destoning. For canning of apricot wholes and halves, 40 Brix sugar syrup was boiled, filtered, and poured into the cans, while for canning of apricot pulp, the pulp obtained was filled into cans. The tin cans were then steam exhausted. After that, the cans were autoclaved for 30 min at 121°C. The cans were then cooled in running tap water to 38°C, cleaned, tagged, and kept at room temperature. Analysis of processed apricot treatments was performed for 12 months at an interval of 4 months.

### Physicochemical Properties

Moisture content, titratable acidity, reducing sugars, total soluble solids, and total sugars of the apricot wholes, halves, and pulp were performed using the standard protocols of AOAC (40). The results were represented as follows: titratable acidity as % malic acid per 100 g, reducing sugars as % glucose, and total soluble solids as degrees Brix (°Bx). Ascorbic acid (AA) content was estimated based on the oxidation of AA by 2, 6-dichlorophenol indophenol dye, and the result was represented as mg per 100 g FW (41).

### Mineral Composition

Minerals were extracted from the apricot treatments by the dry ashing process as elucidated by Sarkar et al. (9) with slight modifications. We determined potassium, phosphorous, zinc, copper, iron, and manganese. Digestion of the sample (2 g) was carried out in a di-acid blend (HNO_3_:HClO_4_, 5:1, v/v). The digested material was dissolved in double-distilled water, which was then filtered using Whatman no. 42. Finally, the volume was made up to 50 ml before subjecting it to mineral estimation. Mineral profile analysis of apricot wholes, halves, and pulp was assessed using Atomic Absorption Spectrophotometer (Labtronics, Model LT-2100), and the absorbance was measured at a wavelength of 766.5 nm (Potassium), 880 nm (Phosphorous), 324.8 nm (Copper), 248.3 nm (Iron), 279.5 nm (Manganese), and 213.9 nm (Zinc). The results were represented as mg/100 g FW.

### RP-HPLC Analysis

#### Extraction

A sample weight (500 g) of each apricot treatment was taken. Methanol (80% v/v) was used as an extraction solvent. Extraction was performed three times with a sample to solution ratio of 1:3. The extracts were then filtered and pooled together. In a rotary vacuum evaporator, the pooled extract was concentrated at 40°C. Prior to HPLC analysis, the concentrate was kept in a desiccator at a low temperature.

#### Analysis

The phenolic compounds of apricot wholes, halves, and pulp were identified and quantified using reversed-phase high-performance liquid chromatography (RP-HPLC) equipment (Agilent Technologies, 1,260 Infinity, Germany) equipped with a manual injector, degasser, quaternary pump, and a diode array detector. In HPLC grade methanol solvent, concentrated extracts of all the treatments [1.0% solution (w/v)] were prepared and filtered through Millipore membrane filters (0.22 mm) and Whatman filter paper no. 42; 20 μl of sample volume was loaded into the C-18 column (Agilent Eclipse Plus, C18 3.5 μm, 4.6 × 100 mm, USUXR16700, USA), which was thermostatically operated at 35°C. The mobile phase consisted of 0.1% formic acid in methanol and H_2_O, and the HPLC gradient was as follows: 5 to 15% in 15 min, 15 to 30% in 20 min, 30 to 40% in 5 min, 40 to 50% in 10 min, 50 to 60% in 5 min, 60 to 75% in 5 min, and finally reaching 95% in a total time of 65 min employed at a flow rate of 1.0 ml/min. Detection and quantification were carried out at 280 nm (for kaempferol, quercetin-3-glucoside, epicatechin, and procyanidin B_2_) and 320 nm (for ellagic acid, gallic acid, chlorogenic acid, and neochlorogenic acid). The retention time and the characteristic UV spectra of the standard compounds were used to identify the phenolic compounds. Results were reported as milligram per kilogram (mg/kg FW). The protocol was followed as elucidated by Wani et al. (42).

#### Total Phenolic Content

The total phenolic content (TPC) of treatments was assayed following the protocol as described by Wani et al. (43), and the content was evaluated using the Folin–Ciocalteu colorimetric assay. Absorbance readings were taken at 725 nm using a spectrophotometer (UV-2450, Shimadzu, Japan), and the results were represented as GAE mg/100 g of FW.

#### Antioxidant Properties (DPPH, MCA, ABTS, and BCBA)

The 2, 2-diphenyl-1-picrylhydrazyl (DPPH) free radical scavenging activity of apricot treatments was performed as elucidated by Wani et al. (43). On a UV–vis spectrophotometer (UV-2450, Shimadzu, Japan), the absorbance reading was taken at 517 nm, and the radical scavenging activity was reported as percent inhibition as follows:


(1) $$\begin{array}{r} \%\;inhibition\;=\;\left[\left(AB\;-\;AA\right)\;/AB\right]\;\times \;100 \end{array}$$

where AA = absorbance after sample incubation and AB = absorbance before sample addition.

The metal chelating activity (MCA) of apricot treatments was assessed using the protocol of Sharma and Gujral (44). On a UV–vis spectrophotometer (UV-2450, Shimadzu, Japan), the absorbance reading was taken at 562 nm, and the percentage chelation of iron (Fe^2+^) was used to determine the metal chelation activity as follows:


(2) $$\begin{array}{l} \begin{array}{c} Iron\;(Fe^{2+})\;chelating\;activity\;(\%)\;=\;[(AB\;-\;AA)\;/AB] \end{array}\\ \;\begin{array}{r} \begin{array}{c} \times \;100 \end{array} \end{array} \end{array}$$

where AA = absorbance after sample incubation and AB = absorbance before sample addition.

The 2, 2-azino-bis-3-ethylbenzothiazoline-6-sulfonic acid (ABTS^+^) radical cation method of apricot treatments was performed following the protocol illustrated by Tembo et al. (45). On a UV–vis spectrophotometer (UV-2450, Shimadzu, Japan), the absorbance reading was taken at 734 nm, and % inhibition was computed as follows:


(3) $$\begin{array}{r} \%\;inhibition\;=\;\left(A_{0}-\;A_{1}\right)\;/A_{0}\times \;100 \end{array}$$

where A0 = absorbance of the control and A1 = absorbance of the sample.

The β-carotene bleaching assay (BCBA) of apricot treatments was performed by measuring the coupled autoxidation of linoleic acid and β-carotene following the protocol of Ueno et al. (46). On a UV–vis spectrophotometer (UV-2450, Shimadzu, Japan), the absorbance reading was taken at 460 nm, and % inhibition of bleaching was computed as follows:


(4) $$\begin{array}{l} \begin{array}{c} \%\;inhibition\;=\;Absorbance\;after\;2\;h\;of\;assay/ \end{array}\\ \begin{array}{c} \;Initial\;absorbance\;\times \;100 \end{array} \end{array}$$

Trolox was used as the reference standard for all the AOX properties, and the results were expressed as μg Trolox equivalent/g DW.

### Statistical Analysis

All the experiments were carried out in triplicates, and the mean values were calculated. Data were assessed statistically by one-way ANOVA using SPSS, Version 22 (IBM Statistics 21.0, Chicago, IL, USA). To measure the significant differences between sample means, Duncan's test was used at 5% level of significance.

## Results

### Physicochemical Properties

The effect of canning and storage on physicochemical characteristics of apricot treatments (wholes, halves, and pulp) is given in Table 1. The data revealed that moisture content varied non-significantly among the apricot treatments throughout the storage period. However, AA, titratable acidity, total sugars, reducing sugars, and total soluble solids (TSS) significantly varied across the treatments. TSS was found highest in canned apricot pulp (37.15 °Bx) followed by apricot wholes (35.59 °Bx) and apricot halves (32.12 °Bx) at 0 month of storage. Apricot pulp showed the highest titratable acidity (1.39%) followed by apricot wholes (1.14%), whereas apricot halves had the lowest titratable acidity (0.81%) at 0 month of storage. In case of sugars, apricot pulp had the highest total sugars (20.74%) and lowest reducing sugars (5.74%) followed by apricot wholes and halves at 0 month of storage. AA content was noticed highest in apricot pulp (7.21 mg/100 g FW) followed by apricot wholes (6.9 mg/100 g FW) and lowest in apricot halves (5.39 mg/100 g FW) at 0 month of storage. The data further revealed that TSS, reducing sugars, and total sugars inclined in all three treatments of apricot during 12 months of storage significantly (*p* < 0.05); however, a decline in titratable acidity and AA was noticed during 12 months of storage in all three forms of apricot treatments significantly (*p* < 0.05).

**Table 1:** Effect of canning and storage on physiochemical properties of canned apricot wholes, halves and pulp.

**Storage** **period**	**Treatments**	**Moisture** **content (%)**	**Total soluble** **solids (°Bx)**	**Titratable** **acidity** **(% malic acid)**	**Total sugars (%)**	**Reducing** **sugars** **(% glucose)**	**Ascorbic acid** **(mg/100g FW)**
0 Month	Wholes	78.36 ± 0.40^Aa^	35.59 ± 0.52^Bc^	1.14 ± 0.15^Bfg^	19.49 ± 0.33^Bb^	9.25 ± 0.20^Ce^	6.9 ± 0.19^Bg^
	Halves	79.56 ± 0.49^Aa^	32.12 ± 0.45^Aa^	0.81 ± 0.07^Aab^	18.17 ± 0.38^Aa^	8.55 ± 0.19^Bd^	5.39 ± 0.17^Acd^
	Pulp	81.65 ± 0.51^Aa^	37.15 ± 0.56^Cd^	1.39 ± 0.18^Bh^	20.74 ± 0.34^Cc^	5.74 ± 0.17^Aa^	7.21 ± 0.24^Bh^
4 Months	Wholes	80.77 ± 0.49^Aa^	36.87 ± 0.42^Bd^	0.92 ± 0.13^Ade^	22.78 ± 0.35^Ad^	11.08 ± 0.13^Cg^	5.58 ± 0.10^Bde^
	Halves	82.54 ± 0.59^Aa^	33.24 ± 0.49^Ab^	0.75 ± 0.15^Acd^	22.46 ± 0.50^Ade^	9.18 ± 0.14^Be^	5.12 ± 0.11^Ab^
	Pulp	83.67 ± 0.66^Aa^	38.26 ± 0.58^Ce^	1.23 ± 0.14^Bgh^	23.65 ± 0.41^Be^	6.78 ± 0.17^Ab^	6.30 ± 0.16^Cf^
8 Months	Wholes	82.87 ± 0.54^Aa^	38.30 ± 0.50^Be^	0.89 ± 0.12^Ade^	26.00 ± 0.40^Af^	12.28 ± 0.13^Ch^	5.19 ± 0.17^Bbc^
	Halves	84.93 ± 0.67^Aa^	35.79 ± 0.55^Ac^	0.65 ± 0.11^Bab^	25.78 ± 0.44^Af^	10.03 ± 0.21^Bf^	4.74 ± 0.16^Aa^
	Pulp	85.38 ± 0.72^Aa^	40.48 ± 0.63^Cf^	1.06 ± 0.12^Bef^	27.47 ± 0.57^Bg^	7.37 ± 0.16^Ac^	5.70 ± 0.19^Ce^
12 Months	Wholes	83.80 ± 0.66^Aa^	38.31 ± 0.53^Be^	0.83 ± 0.14A^bcd^	29.84 ± 0.52^Bh^	13.33 ± 0.20^Ci^	4.54 ± 0.16^Aa^
	Halves	85.25 ± 0.73^Aa^	37.04 ± 0.58^Ad^	0.59 ± 0.12^ABa^	27.58 ± 0.56^Ag^	11.35 ± 0.15^Bg^	4.51 ± 0.20^Ba^
	Pulp	86.77 ± 0.75^Aa^	41.22 ± 0.62^Cf^	1.00 ± 0.15^Bfg^	33.95 ± 0.64^Ci^	8.45 ± 0.19^Ad^	5.12 ± 0.18^Cbc^

*All values are mean ± standard deviation of three replicates. Means in the same column with different upper case superscripts differ significantly (p ≤ 0.05) Means with different lower case superscripts in the same column (storage months) indicate significant differences (p ≤ 0.05)*.

### Mineral Composition

The influence of canning and storage on the mineral composition of apricot wholes, halves, and pulp is summarized in Table 2. The data indicated that canning affected the mineral composition of all the treatments of apricot significantly (*p* < 0.05). In this study, apricot pulp had the highest potassium content (1,508 mg/100 g FW), while apricot halves had the lowest potassium content (1,377 mg/100 g FW) at 0 month of storage. Iron content was found highest in apricot pulp (9.01 mg/100 g FW), while it was found lowest in apricot halves (3.02 mg/100 g FW). In contrast, phosphorous and copper were found highest in apricot halves (51.84 mg/100 g FW; 2.84 mg/100 g FW), respectively. Zinc and manganese were noticed highest in apricot wholes (19.21 mg/100 g FW and 0.79 mg/100 g FW, respectively) at 0 month of storage. However, the data further revealed that mineral content decreased in all the treatments during the 12 months of storage period significantly (*p* < 0.05).

**Table 2:** Effect of canning and storage on mineral composition of apricot wholes, halves and pulp.

**Storage** **period**	**Treatments**	**Potassium**	**Phosphorous**	**Zinc**	**Copper**	**Iron**	**Manganese**
0 Month	Wholes	1439 ± 5.00^Bb^	46.18 ± 2.23^Bb^	19.21 ± 0.44^Bc^	2.72 ± 0.56^Aa^	8.96 ± 0.59^Bb^	0.79 ± 0.17^Bc^
	Halves	1377 ± 4.00^Aa^	51.84 ± 2.15^Cc^	10.71 ± 0.31^Aa^	2.84 ± 0.63^Aa^	3.02 ± 0.01^Aa^	0.63 ± 0.14^ABab^
	Pulp	1508 ± 5.00^Cd^	30.76 ± 1.84^Aa^	10.52 ± 0.37^Aa^	2.72 ± 0.52^Aa^	9.01 ± 0.61^Bb^	0.49 ± 0.11^Aab^
4 Months	Wholes	1438 ± 5.00^Bb^	45.77 ± 2.00^Bb^	18.97 ± 0.79^Bc^	2.65 ± 0.21^Aa^	8.98 ± 0.37^Bb^	0.77 ± 0.25^Ac^
	Halves	1375 ± 3.00^Aa^	50.92 ± 2.42^Cc^	10.24 ± 0.52^Aa^	2.81 ± 0.33^Aa^	3.05 ± 0.02^Aa^	0.62 ± 0.23^Abc^
	Pulp	1506 ± 5.00^Cd^	30.14 ± 1.84^Aa^	10.21 ± 0.56^Aa^	2.69 ± 0.27^Aa^	9.07 ± 0.32^Bb^	0.46 ± 0.16^Aab^
8 Months	Wholes	1436 ± 4.00^Bb^	45.13 ± 2.68^Bb^	18.14 ± 0.79^Bbc^	2.64 ± 0.35^Aa^	9.13 ± 0.46^Bb^	0.76 ± 0.22^Bc^
	Halves	1373 ± 3.00^Aa^	50.37 ± 2.66^Cc^	10.23 ± 0.63^Aa^	2.79 ± 0.31^Aa^	3.08 ± 0.04^Aa^	0.61 ± 0.14^ABab^
	Pulp	1502 ± 4.00^Cd^	29.43 ± 1.72^Aa^	10.15 ± 0.58^Aa^	2.66 ± 0.26^Aa^	9.09 ± 0.42^Bb^	0.45 ± 0.13^Aa^
12 Months	Wholes	1434 ± 4.00^Bb^	44.98 ± 2.14^Bb^	17.93 ± 0.65^Bb^	2.61 ± 0.32^Aa^	9.17 ± 0.42^Bb^	0.74 ± 0.18^Bbc^
	Halves	1371 ± 3.00^Aa^	49.72 ± 2.96^Cc^	10.14 ± 0.54^Aa^	2.75 ± 0.37^Aa^	3.12 ± 0.04^Aa^	0.59 ± 0.12^ABab^
	Pulp	1495 ± 4.00^Cc^	29.21 ± 1.72^Aa^	10.03 ± 0.49^Aa^	2.62 ± 0.28^Aa^	9.13 ± 0.35^Bb^	0.42 ± 0.15^Aa^

*All values are mean ± standard deviation of three replicates. Means in the same column with different upper case superscripts differ significantly (p ≤ 0.05). Means with different lower case superscripts in the same column (storage months) indicate significant differences (p ≤ 0.05)*.

### Phenolic Composition

The amount of phenolic compounds differ significantly among the three apricot treatments (wholes, halves, and pulp) throughout the storage period. HPLC detected eight phenolic compounds in all the apricot treatments, namely, chlorogenic acid, neochlorogenic acid, quercitin-3-glucoside, gallic acid, kaempferol, ellagic acid, procyanidin B_2_, and epicatechin (Table 3). The data indicated that across the treatments, the content of chlorogenic acid (34.45 mg/kg FW), neochlorogenic acid (26.52 mg/kg FW), quercitin-3-glucoside (16.78 mg/kg FW), gallic acid (5.37 mg/kg FW), kaempferol (14.22 mg/kg FW), ellagic acid (6.02 mg/kg FW), procyanidin B_2_ (8.80 mg/kg FW), and epicatechin (9.87 mg/kg FW) was observed to be significantly higher in apricot pulp followed by apricot wholes and halves at 0 month of storage. The data further revealed that phenolic composition was observed to be declined significantly (*p* < 0.05) during 12 months of storage in all the treatments.

**Table 3:** Effect of canning and storage on phenolic composition (mg/kg FW) of apricot wholes, halves and pulp.

**Storage** **period**	**Treatments**	**Chlorogenic** **acid**	**Neochlorogenic** **acid**	**Quercitin-3-** **glucoside**	**Gallic acid**	**Kaempferol**	**Ellagic acid**	**Procyanidin** **B**_**2**_	**Epicatcehin**
0 Month	Wholes	31.86 ± 0.26^Bh^	21.77 ± 0.25^Bh^	13.03 ± 0.17^Bg^	4.12 ± 0.14^Bg^	12.12 ± 0.14^Bh^	5.28 ± 0.17^Ag^	7.24 ± 0.13^Bg^	.63 ± 0.14^Be^
	Halves	25.11 ± 0.14^Ad^	19.31 ± 0.19^Af^	10.03 ± 0.15^Ad^	3.62 ± 0.11^Af^	11.78 ± 0.13^Ag^	5.12 ± 0.14^Ag^	6.12 ± 0.14^Ae^	6.73 ± 0.13^Acd^
	Pulp	34.45 ± 0.29^Cj^	26.52 ± 0.23^Ck^	16.78 ± 0.18^Ck^	5.37 ± 0.19^Cj^	14.22 ± 0.11^Ck^	6.02 ± 0.11^Bh^	8.80 ± 0.19^Ci^	9.87 ± 0.16^Cf^
4 Months	Wholes	30.75 ± 0.21^Bg^	20.61 ± 0.17^Bg^	12.54 ± 0.19^Bf^	3.66 ± 0.12^Bf^	10.31 ± 0.12^Be^	4.53 ± 0.13^Af^	6.58 ± 0.14^Bf^	7.12 ± 0.13^Bde^
	Halves	21.62 ± 0.17^Ac^	16.16 ± 0.15^Ad^	8.85 ± 0.14^Ab^	3.05 ± 0.13^Ae^	9.32 ± 0.09^Ad^	4.32 ± 0.12^Ae^	5.09 ± 0.14^Ac^	5.62 ± 0.14^Abc^
	Pulp	33.11 ± 0.28^Ci^	26.21 ± 0.23^Ck^	16.14 ± 0.12^Cj^	5.14 ± 0.12^Ci^	13.31 ± 0.12^Cj^	5.15 ± 0.15^Bg^	7.68 ± 0.17^Ch^	8.41 ± 0.13^Cef^
8 Months	Wholes	27.12 ± 0.18^Be^	18.55 ± 0.16^Be^	10.71 ± 0.13^Be^	2.90 ± 0.07^Bd^	9.14 ± 0.16^Bd^	3.96 ± 0.09^Bd^	5.39 ± 0.15^Bd^	6.62 ± 0.11^Bcd^
	Halves	19.5 ± 0.15^Ab^	14.40 ± 0.12^Ab^	9.71 ± 0.11^Ac^	2.17 ± 0.09^Ab^	8.18 ± 0.11^Ab^	3.09 ± 0.08^Ac^	4.00 ± 0.13^Ab^	4.73 ± 0.13^Aab^
	Pulp	31.61 ± 0.26^Ch^	25.13 ± 0.18^Cj^	15.71 ± 0.14^Ci^	4.82 ± 0.12^Ch^	12.63 ± 0.14^Ci^	4.13 ± 0.14^Cd^	6.17 ± 0.15^Ce^	7.20 ± 0.15^Cde^
12 Months	Wholes	25.21 ± 0.22^Bd^	15.38 ± 0.16^Bc^	9.85 ± 0.19^Bcd^	2.50 ± 0.08^Bc^	8.73 ± 0.12^Bc^	2.76 ± 0.07^Bb^	4.05 ± 0.13^Bb^	5.20 ± 0.14^Bab^
	Halves	17.1 ± 0.17^Aa^	12.9 ± 0.17^Aa^	6.14 ± 0.11^Aa^	1.60 ± 0.04^Aa^	7.02 ± 0.14^Aa^	2.25 ± 0.06^Aa^	2.90 ± 0.08^Aa^	3.66 ± 0.09^Aa^
	Pulp	29.61 ± 0.24^Cf^	23.6 ± 0.24^Ci^	15.43 ± 0.14^Ch^	4.16 ± 0.13^Cg^	10.93 ± 0.19^Cf^	3.71 ± 0.14^Cd^	5.15 ± 0.13^Cc^	6.00 ± 0.12^Cab^

*All values are mean ± standard deviation of three replicates. Means in the same column with different upper case superscripts differ significantly (p ≤ 0.05). Means with different lower case superscripts in the same column (storage months) indicate significant differences (p ≤ 0.05)*.

### Total Phenolic Content and Antioxidant Properties (DPPH, ABTS, MCA, and BCBA)

Table 4 presented the TPC and AOX properties of apricot wholes, halves, and pulp, namely, DPPH radical scavenging activity, MCA, ABTS^+^, and BCBA. TPC and AOX capacity (AC) varied significantly (*p* < 0.05) among the studied apricot treatments. The data indicated that TPC was observed highest in apricot pulp (13.76 GAE mg/100 g FW) followed by apricot wholes (8.09 GAE mg/100 g FW), while the lowest was noticed in apricot halves (6.48 GAE mg/100 g FW) at 0 month of storage. Apricot pulp (92.23 TEAC μg/g DW) exhibited the highest AC (DPPH) followed by apricot wholes (88.16 TEAC μg/g DW) and apricot halves (77.37 TEAC μg/g DW). Highest AC (ABTS^+^) was noticed in apricot pulp (92.33 TEAC μg/g DW), while the lowest AC was noticed in apricot halves (84.21 TEAC μg/g DW). Apricot pulp (33.80 TEAC μg/g DW) showed the highest AC (MCA) followed by apricot wholes (11.97 TEAC μg/g DW) and apricot halves (10.54 TEAC μg/g DW). Highest AC (BCBA) was observed in apricot pulp (68.40 TEAC μg/g DW), while the lowest AC was observed in apricot halves (TEAC 50.63 μg/g DW) at 0 month of storage. However, TPC and AOX properties were observed to be declined significantly (*p* < 0.05) during 12 months of storage in all the treatments.

**Table 4:** Effect of canning and storage on total phenolic content and antioxidant properties of apricot wholes, halves and pulp.

**Storage** **period**	**Treatments**	**TPC** **(GAE mg/100 g^−1^** **FW)**	**DPPH** (**TEAC μg g^−1^ DW)**	**ABTS** **(TEAC μg g^−1^ DW)**	**Metal Chelation** **(TEAC μg g^−1^ DW)**	**BCBA** **(TEAC μg g^−1^ DW)**
0 Month	Wholes	8.09 ± 0.17^Bc^	88.16 ± 2.25^Bbc^	87.35 ± 2.69^ABde^	11.97 ± 0.44^Ae^	62.86 ± 1.93^Bd^
	Halves	6.48 ± 0.14^Ab^	77.57 ± 1.58^Aa^	84.21 ± 2.26^Abc^	10.54 ± 0.89^Ade^	50.63 ± 1.26^Ac^
	Pulp	13.76 ± 0.23^Cg^	92.23 ± 2.97^Ce^	92.33 ± 2.15^Be^	33.80 ± 1.47^Bh^	68.40 ± 1.19^Cf^
4 Months	Wholes	8.81 ± 0.15^Be^	88.14 ± 2.36^Bbc^	85.41 ± 2.24^Acd^	9.75 ± 0.26^Acd^	52.32 ± 1.13^Bc^
	Halves	6.32 ± 0.11^Aab^	77.03 ± 1.44^Aa^	80.88 ± 2.53^Abc^	9.12 ± 0.18^Abc^	46.41 ± 0.84^Ab^
	Pulp	13.55 ± 0.26^Cg^	91.80 ± 2.36^Bde^	91.32 ± 2.69^Be^	31.64 ± 1.41^Bg^	67.08 ± 1.26^Cef^
8 Months	Wholes	8.69 ± 0.14^Bde^	87.30 ± 2.46^Bbc^	84.67 ± 2.94^ABcd^	8.54 ± 0.12^Abc^	52.57 ± 0.63^Bc^
	Halves	6.25 ± 0.13^Aab^	76.13 ± 1.36^Aa^	80.05 ± 2.41^Aab^	6.90 ± 0.95^Ab^	41.77 ± 1.26^Aa^
	Pulp	13.12 ± 0.21^Cf^	91.25 ± 2.48^Bcd^	89.93 ± 2.69^Be^	31.17 ± 1.88^Bg^	65.87 ± 1.10^Ce^
12 Months	Wholes	8.38 ± 0.16^Bcd^	86.69 ± 2.36^Bb^	84.11 ± 2.97^Bcd^	7.54 ± 0.47^Bb^	51.89 ± 1.26^Bc^
	Halves	6.09 ± 0.12^Aa^	75.66 ± 1.49^Aa^	77.10 ± 1.66^Aa^	4.58 ± 0.42^Aa^	40.92 ± 0.73^Aa^
	Pulp	12.97 ± 0.19^Cf^	90.52 ± 2.65^Bcd^	88.18 ± 2.11^Bde^	26.21 ± 1.15^Cf^	65.63 ± 1.30^Ce^

*All values are mean ± standard deviation of three replicates*.

*Means in the same column with different upper case superscripts differ significantly (p ≤ 0.05)*.

*Means with different lower case superscripts in the same column (storage months) indicate significant differences (p ≤ 0.05)*.

*TPC, Total phenolic content, DPPH, 2, 2–diphenyl-1-picrylhydrazyl, ABTS, 2, 2-azino-bis-3-ethylbenzothiazoline-6-sulfonic acid, BCBA, β-carotene bleaching assay*.

## Discussion

### Physicochemical Properties

The moisture content varied non-significantly across the treatments. However, the highest TSS was noticed in canned apricot pulp followed by apricot wholes and apricot halves at 0 month of storage. The highest TSS in canned apricot pulp is likely due to the conversion of complex substances into soluble forms at higher temperatures which are retained in the pulp (28). Further, TSS was positively correlated with total sugars. However, TSS was increased significantly in all three treatments of apricot during 12 months of storage possibly due to the hydrolysis of complex carbohydrates into simpler ones during storage (47). The findings obtained in this research were corroborated to the results of canned apricots by Wani et al. (28). The titratable acidity of the fruit is directly related to its organic acid content. During storage and ripening, its concentration usually reduces due to enzyme-catalyzed processes, which makes the fruit taste sweeter (41). Organic acids are also commonly utilized as preservatives in a variety of fruit products. The results for titratable acidity showed the highest value for the apricot pulp followed by apricot wholes and apricot halves at 0 month of storage. The highest titratable acidity value of apricot pulp may be because of the delaying of the ripening process due to pulping. However, a decline in titratable acidity was noticed during 12 months of storage in all three forms of apricot treatments significantly (*p* < 0.05). This could be because organic acids are used in respiratory metabolism during storage (48). Vijayanand et al. (49) also found that after 2 months of storage at ambient temperature, the acidity of canned mango slices decreased gradually.

Sugars are a vital component of the diet and a quick energy source for the body. Fruit with elevated sugar content can also be used to determine its maturity (2). The results depicted that at 0 month of storage, apricot pulp showed the highest total sugars and lowest reducing sugars. Sugar utilization in browning reactions may account for the lowest reducing sugars in apricot pulp (28). However, reducing sugars and total sugars enhanced in all three treatments of apricot during 12 months of storage significantly. This increase in reducing sugars may be because of the breakdown of complex carbohydrates and non-reducing sugars, while the breakdown of complex carbohydrates into simpler sugars may be the reason for the increase in total sugars (28). Vijayanand et al. (49) found a comparable rise in reducing sugars over a 2-month storage period in canned mango slices. Gowda and Huddar (50) observed a similar rise in total sugars during storage in canned mango slices.

Ascorbic acid is known as a potent free radical trapping agent because it prevents the degradation of fruit during ripening. Apart from that, AA provides an indication of the nutritional quality of F&Vs (41). According to several studies, AA is an extremely heat-labile compound that is readily destroyed when exposed to heat (45). Thus, the influence of canning and storage on the AA content of apricot wholes, halves, and pulp was evaluated. The data revealed that the AA content was noticed highest in the apricot pulp. Higher solute concentrations in apricot pulp, particularly sugars and organic acids, may protect against AA oxidation by chelating metal ions and lowering their catalytic efficacy (51). However, AA was noticed to be declined in all three treatments of apricot during 12 months storage period significantly. The higher degradation of AA in all the treatments was due to higher storage temperatures. Further, because enzymatic degradation is eliminated during processing at higher temperatures, non-enzymatic mechanisms could be the reason for AA loss during 12 months storage period (45). The formation of dicarbonyl compounds and dehydroascorbic acid during its degradation (diketogulonic acid, xylosone, 3-deoxythreosone, and erythrulose) can experience AA browning (non-enzymatic browning) through a Strecker-like decomposition process with amino acids resulting in brown pigments formation (51), including hydroxymethylfurfural. These results coincide well with the results recorded by Cao et al. (52) for cucumber.

### Mineral Composition

Canning affected the mineral composition of all the treatments significantly. Copper, iron, zinc, and manganese were found in lesser quantities in all the treatments and were classified as microelements, while phosphorous and potassium levels were much higher and were classified as macroelements. In this study, we found that among the macrominerals, potassium content was found highest in all three treatments at 0 month of storage. These values were comparable with the previous reports on mineral analysis conducted by Akin et al. (29) on various apricot varieties. The data further revealed that potassium and iron were found highest in apricot pulp, phosphorous and copper in apricot halves, and zinc and manganese in apricot wholes at 0 month of storage. Minerals are not affected as much due to canning as minerals are thermally stable during normal processing conditions. However, minerals decreased during the storage period due to the leaching of minerals to the syrup, while the concentration of iron gradually increased with storage of canned treatments which may be attributed to the possible deposition of iron from the iron lids used for canning. These results are in line with the previous results recorded by Rickman et al. (53) for canned F&Vs. Hence, the apricot pulp could contribute to high minerals than that of apricot wholes and halves.

### Phenolic Composition

The RP-HPLC analysis was performed in order to identify major polyphenol compounds and account for the elevated AC and TPC values noticed in apricot wholes, halves, and pulp. At 0 month of storage, the content of chlorogenic acid, neochlorogenic acid, quercitin-3-glucoside, gallic acid, kaempferol, ellagic acid, procyanidin B_2_, and epicatechin was observed to be significantly higher in apricot pulp followed by apricot wholes and halves. Canning of apricot pulp was carried out without a medium, and thus, the polyphenolic components that would have leached into the canning medium were retained in the slurry. It can, therefore, be envisaged that chlorogenic acid, quercitin-3-glucoside, gallic acid, neochlorogenic acid, kaempferol, ellagic acid, procyanidin B_2_, and epicatechin contribute significantly to the AC and TPC found in the apricot pulp. The results were comparable with Wani et al. (42) for canned apricot pulp. Hence, these AOX phytochemicals found in apricot pulp could be an essential parameter for consumers, as they play a key role in the detoxification of ROS in the human body and in combating anti-aging and many degenerative diseases (9). However, these polyphenolic compounds were observed to be declined significantly during 12 months of storage in all the treatments. According to Chen et al. (54), processing at higher temperatures can result in complex chemical and physical reactions that affect phenolic profile, such as the liberation of bound phenolic substances, polyphenols decomposition, and the breakdown and conversion of phenolic substances. Earlier, Rababah et al. (37) investigated that storing apricot jam at 25°C for 5 months resulted in a significant loss in polyphenols. Aaby et al. (35) reported a 20% decline in ellagitannin and 17% decline in ellagic acid derivatives during the processing of strawberries. Odriozola-Serrano et al. (39) noticed that the concentration of quercetin and kaempferol in strawberry juice is unaffected by thermal pasteurization (90°C, 60 s); however, Igual et al. (36) found that the concentration of quercetin, naringenin, naringin, and narirutin in grapefruit juice is reduced.

### Total Phenolic Content

The TPC varied significantly across the treatments. The data revealed that TPC was observed highest in apricot pulp followed by wholes and halves at 0 month of storage. Higher phenolic content in apricot pulp is attributed to factors such as increased leaching of soluble chemicals and the liberation of phenolics from their bonded forms to the pulp due to cell injury (28). The findings were corroborated to the findings of Stevanato et al. (55). However, during 12 months of storage, TPC was declined significantly in all three apricot treatments. Polyphenols interacting with sugars and sugar metabolites could be the possible justification behind a reduction in TPC during storage (56). It is also likely that complex formation occurs between phenolic substances and proteins resulting in changes in the nutritional, functional, and structural attributes of both substances. The association may be because the phenolic group is a good hydrogen donor, forming hydrogen bonds with the carboxyl group of the protein (45). Furthermore, higher storage temperatures can cause phenolic compounds to undergo chemical oxidation, resulting in the formation of polymers and quinones (57), thereby resulting in a decrease in phenolic compounds during storage. Similar reports were demonstrated by Wani et al. (28) for dried, frozen, and canned apricots.

### Antioxidant Properties (DPPH, ABTS^+^, MCA, and BCBA)

Preservation techniques are expected to be the reason for the significant loss of natural AOXs. When compared with fresh fruits, processed fruit items are considered to provide lesser health-promoting protection, and yet, the functional characteristics of the latter may remain stable throughout the storage (45). The effect of canning and storage period on AOX stability was studied in apricot wholes, halves, and pulp during 12 months of storage. Four techniques (MCA, DPPH, BCBA, and ABTS^+^) were employed due to numerous reaction features and processes involved in a complex or mixed system (58, 59) in order to reflect the overall AOX potential of apricots. For instance, ABTS^+^ measures both lipophilic and hydrophilic AOXs, while the DPPH method only considers lipophilic AOXs (58). The BCBA is among the most widely used method for measuring AOX activity in the area of food chemistry (46).

Apricot pulp exhibited the highest DPPH radical scavenging potential followed by apricot wholes and apricot halves at 0 month of storage. The findings of DPPH scavenging potential are in line with the ABTS^+^, MCA, and BCBA indicating the maximum AC of apricot pulp followed by apricot wholes and apricot halves. Generally, treatments with high TPCs exhibited higher DPPH scavenging activity, whereas chelating agents can prevent the peroxidation of lipids and suppress the generation of free radicals by deactivating the transition metals (2). As expected, apricot pulp exhibited the highest AOX properties (DPPH, ABTS^+^, and MCA), which may be due to its higher AA and phenolic compounds. Besides this, non-enzymatic browning occurs during the processing of pulp, which may further contribute to its higher AOX properties (57). AA browning is considered to be the cause of non-enzymatic browning of pulp during thermal processing or storage (51). Similar reports were demonstrated by Bof et al. (60) for the AOX potential of fruit pulp and jelly. AA and phenolics showed higher AOX activity in *Amaranthus hypochondriacus* (61), *Amaranthus tricolor* (62), *Amaranthus blitum* (63), weedy species (64), stem amaranth (65), green morph amaranth (66), and red morph amaranth (67), which are corroborative to the present findings. Furthermore, apricot pulp significantly (*p* < 0.05) interrupted the β-carotene bleaching in comparison with apricot wholes and halves. Discoloration of β-carotene occurs in the absence of AOXs because it binds with linoleic acid and develops free radicals. As a result, the β-carotene bleaching rate can be delayed in the presence of AOXs (2). Several studies investigated that the BCBA is linked to flavonoids and polyphenols components, which can prevent linoleic acid oxidation and the generation of hydroperoxides (68, 69), thereby supporting our results. However, the AOX activity decreased during 12 months of storage owing to the diffusion of hydrophilic compounds, including AOXs and phenolics, to syrup (70). Furthermore, depletion in AOX activity noticed during storage could be related to a decline in thermally susceptible phenolic compounds and vitamin C and the generation of melanoidins with pro-oxidant characteristics (71). Similar reports were demonstrated by Tembo et al. (45) for baobab fruit pulp.

## Conclusion

Apricot fruit is rich in AA, organic acids, and phenolic compounds, which can be utilized in a variety of food and pharmaceutical applications. This study revealed that eight polyphenolic compounds were detected by RP-HPLC in all the treatments and were declined throughout the storage period. AOX properties (DPPH, ABTS^+^, MCA, and BCBA) and TPC were investigated, which were found higher in apricot pulp throughout the storage period. Furthermore, apricot pulp exhibited excellent sources of nutrients and minerals compared with apricot wholes and halved that declined during the storage period. It can be concluded that apricot pulp retained most of its nutrients, minerals, AOX phytochemicals, and AOX activity during canning and 12 months of storage that offered huge prospects for nutritional and health-boosting effects and that consuming it can reduce the danger of numerous oxidative stress-related disorders such as diabetes, cancer, aging, and cardiovascular disorders.

## Data Availability Statement

The original contributions presented in the study are included in the article/supplementary material, further inquiries can be directed to the corresponding authors.

## Author Contributions

NJ: Writing original draft, review, and editing. SA: Investigation. SMW: Conceptualization. SM: Formal Analysis. AM: Supervision. SAW: Critically reviewed the manuscript. DH, RR, and MG: Interpreted the data and analyzed the samples. All authors have read and approved the final version of the manuscript.

## Funding

SMW acknowledges the Department of Biotechnology, GOI for providing the financial support. The authors extend their appreciation to the researchers supporting project number (RSP-2021/393), King Saud University, Riyadh, Saudi Arabia.

## Conflict of Interest

The authors declare that the research was conducted in the absence of any commercial or financial relationships that could be construed as a potential conflict of interest.

## Publisher's Note

All claims expressed in this article are solely those of the authors and do not necessarily represent those of their affiliated organizations, or those of the publisher, the editors and the reviewers. Any product that may be evaluated in this article, or claim that may be made by its manufacturer, is not guaranteed or endorsed by the publisher.
